# A rare case of Alveolar Soft-Part Sarcoma in the Uterine cervix

**DOI:** 10.1186/s13000-025-01620-7

**Published:** 2025-04-02

**Authors:** Mei Du, Yanli Li, Xiaorong Fan, Han Gao, Jie Shi, Shiyu Cheng, Tingzhu Meng

**Affiliations:** 1 Medical College, Wuhan University of Science and Technology, Wuhan, China; 2 Department of Gynecology Hubei Province Maternal and Infant Health Hospital, Tongji Medical College, Huazhong University of Science and Technology, Wuhan, China

**Keywords:** Alveolar soft-part sarcoma, Uterine cervix, Clinicopathological characteristic, Immunophenotype, Prognosis

## Abstract

Alveolar soft-part sarcoma (ASPS), a rare and malignant neoplasm of soft tissues, comprises less than 1% of all soft-tissue sarcomas and is characterized by distinct histopathological and molecular markers. A 27-year-old female presented with a history of postcoital vaginal bleeding and intermittent bleeding over the preceding month. Imaging studies identified abnormal echogenicity and vascular patterns in the posterior cervical lip. Initial histopathological assessment indicated a perivascular epithelioid cell tumor (PEComa) with *TFE3* gene rearrangement; however, subsequent immunohistochemical and molecular analyses corroborated the diagnosis of ASPS. The patient underwent a total laparoscopic hysterectomy with bilateral salpingo-oophorectomy. Postoperative pathology revealed that the residual tumor was confined to the inner third of the cervix, with no evidence of lymphovascular or perineural invasion. The patient did not receive adjuvant therapy and was followed for three months postoperatively, during which no recurrence or metastasis was observed. Given the extreme rarity of ASPS, its diagnosis necessitates meticulous scrutiny by pathologists to inform and guide subsequent therapeutic approaches.

## Introduction

Alveolar soft-part sarcoma (ASPS) is an uncommon malignant tumor that originates in soft tissues, representing less than 1% of all soft tissue sarcomas. The 5-year survival rate for patients with ASPS is around 56% [1]. It is characterized by its epithelioid morphology with pseudoadenoid structures, high vascularity, and the hallmark *ASPSCR1-TFE3* gene fusion, making *TFE3* nuclear expression a critical diagnostic marker. In the 2023 categorization of soft tissue tumors by the World Health Organization (WHO), it remains categorized as a tumor with an undetermined origin [2]. It is an uncommon occurrence, with just approximately 50 documented examples to date. This report presents a case of ASPS that occurred in the uterine cervix and provides a retrospective analysis of it based on existing literature. Significantly, the Periodic Acid-Schiff (PAS) test yielded negative results in this particular case, which contrasts with the normal characteristics of ASPS.

## Case description

A 27-year-old female patient reported experiencing postcoital vaginal bleeding for the past 3 years, along with occasional vaginal bleeding over the last month.A gynecological examination revealed cervical hypertrophy with ectropion and congestion on the anterior lip, while the posterior lip appeared smooth, with no contact bleeding or tenderness. No significant abnormalities were detected in the uterus or bilateral adnexa. Gynecological ultrasonography demonstrated abnormal echogenicity and vascular flow in the posterior cervical lip (reduced echogenicity relative to the anterior lip, measuring 1.7 × 1.4 cm with indistinct borders and a crab-foot-like pattern). Imaging further revealed abundant radial blood flow in the posterior lip, creating a striking hypervascular appearance, characterized by a dense, branching vascular network. Imaging revealed a small cystic lesion in the right adnexal region and fluid accumulation in the pouch of Douglas. Pelvic MRI further suggested a cervical cyst on the anterior lip, with iso-T1 and slightly hyper-T2 signals, approximately 1.2 cm in diameter. The patient subsequently underwent a cervical LEEP (loop electrosurgical excision procedure) to excise the cervical mass. Histopathological examination revealed a perivascular epithelioid cell tumor (PEComa) in the cervix, measuring approximately 2 × 0.9 cm. The tumor exhibited immunophenotypic features suggestive of a *TFE3* gene-rearranged PEComa, with involvement at the stromal margin and no evidence of vascular invasion. Given the unusual and rare pathology, an external expert consultation was sought, concluding that the tumor was a mesenchymal neoplasm with epithelioid morphology in the cervix. Immunohistochemical and molecular analysis favored a diagnosis of Alveolar Soft Part Sarcoma (ASPS), though *TFE3* gene-rearranged PEComa could not be completely excluded. Tumor cells were observed at the cervical margin, with no definitive evidence of vascular invasion.

**Fig. 1:**
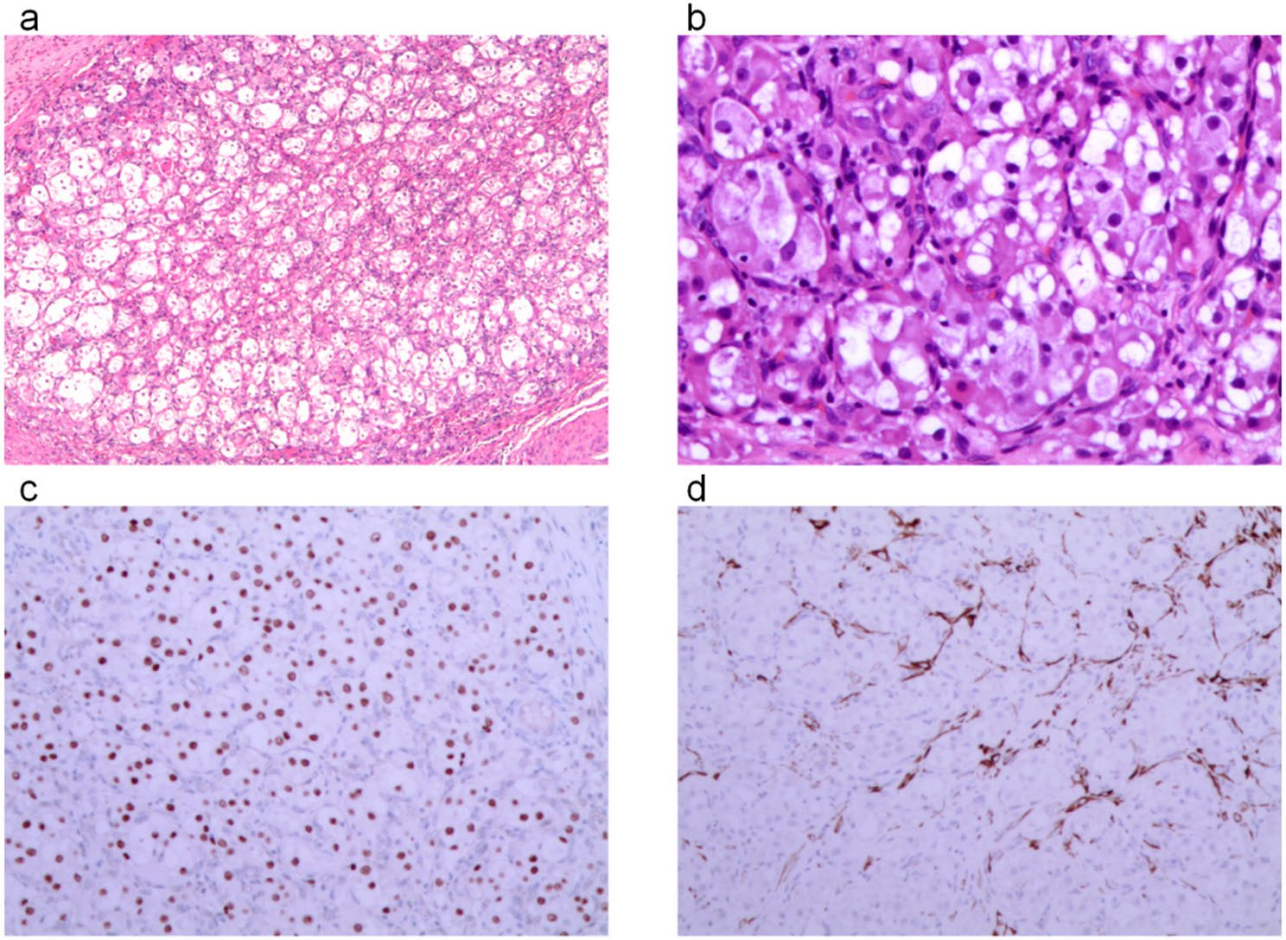
**a**: Tumor cells arranged in solid nests (H&E, 4x); **b**: Tumor cells with epithelioid morphology (H&E, 200x); **c**: Tumor cells showing positive *TFE3* staining (H&E, 100x); **d**: Positive Desmin staining observed in tumor cells (H&E, 100x)

The patient subsequently underwent laparoscopic total hysterectomy with bilateral salpingo-oophorectomy, along with biopsies of the omentum and peritoneum. Postoperative pathology revealed a residual tumor measuring 2 × 1 mm, with invasion of less than one-third of the cervix (less than 3 mm), and no evidence of lymphovascular or perineural invasion. The remaining gynecologic organs, as well as the omental and peritoneal biopsies, were all negative for tumor. The patient did not receive any adjuvant therapy and was monitored for 3 months without any indications of tumor spread or recurrence (Fig. 1).

The tumor cells show an epithelioid morphology with mild atypia, organized into solid nests and alveolar structures. The cytoplasm appears eosinophilic, granular, or clear, with a mitotic rate of 1 per 10 high-power fields (HPF), and there is no noticeable necrosis. This histomorphology is characteristic of ASPS, which can be differentiated from other tumors such as PEComa based on its specific cellular arrangement and immunohistochemical findings. Immunohistochemical analysis revealed positive TFE3 staining in the tumor cells, supporting the diagnosis of ASPS. Desmin positivity was observed in focal areas, which further helped in distinguishing it from other soft tissue tumors.

Immunohistochemical analysis at the original institution showed negative results for MelanA, HMB45, MiTF, and PNL2. Molecular testing also detected the *ASPSCR1-TFE3* gene fusion. Additional immunohistochemical analysis from the consulted institution revealed negative results for epithelial markers PCK and EMA, with focal positivity for smooth muscle markers SMA and Desmin. Other markers including MyoD1, S-100, SOX-10, synaptophysin, CgA, CD31, and D2-40 were negative, with Ki67 showing a proliferative index of approximately 10%. *TFE3* and CD68 were positive, while PAS staining was negative. Immunohistochemical analysis revealed negative results for melanocytic markers MelanA, HMB45, MiTF, and PNL2. Epithelial markers PCK and EMA were also negative. Smooth muscle markers, including SMA and Desmin, showed focal positivity, while MyoD1 was negative. Neuroendocrine markers S-100, SOX-10, synaptophysin, and chromogranin A (CgA) were negative. Vascular markers CD31 and D2-40 were negative. The tumor exhibited approximately 10% positivity for the Ki67 proliferation marker. Kidney-related markers PAX8 and RCC were negative. Special staining showed positive results for *TFE3* and CD68, while PAS staining was negative.

## Discussion

ASPS is an uncommon soft tissue tumor, first described by Christopherson et al. in 1952 [3]. Although ASPS can occur in women, it is more commonly found in soft tissue sites rather than gynecological sites, even in adult women. The disease has a wide age range of onset, from 8 to 68 years, and although it primarily affects adolescents, it can also occur in adults. While ASPS most frequently arises in deep soft tissues, it has been documented in other sites including the uterus, vulva, vagina, mediastinum, stomach, and breast [4]. ASPS is highly vaso-invasive, allowing tumor cells to spread through the bloodstream, frequently metastasizing to distant sites like the lungs, brain, and bones, with a particular propensity for brain metastasis compared to other soft-tissue sarcomas. Despite its metastatic potential, ASPS generally presents as a painless and indolent tumor, often remaining undetected due to its slow-growing nature. Detection typically occurs through imaging modalities such as MRI or CT scans. Cervical ASPS typically presents with symptoms such as abnormal uterine bleeding or postcoital vaginal bleeding, which often leads to early detection. In some cases, preoperative diagnosis is possible through cytological examination, although this is less common. These clinical findings are consistent with previous reports in the literature. Cervical ASPS typically presents with abnormal uterine bleeding, with some cases detected early due to this symptom. In a few instances, the diagnosis can be made preoperatively through cytological examination, as seen in several reported cases [5]. Imaging does not typically reveal the early stages of cervical ASPS.

**Table 1:** Immunohistochemical and genetic testing for cervical ASPS

	TFE3	HMB45	Melan-A	SMA	MyoD1	Desmin	PAS	ASPSCR-TFE3
Roma A A [6]	+	-	NP	-	NP	-	NP	NP
Schoolmeester J K [6]	+	-	-	-	NP	-	NP	+
Schoolmeester J K [6]	+	-	-	-	NP	-	NP	+
Huang S W [7]	+	-	-	+	NP	-	+	-
Lee H J [8]	-	-	NP	+	+C	-	NP	NP
	TFE3	HMB45	Melan-A	SMA	MyoD1	Desmin	PAS	ASPSCR-TFE3
Feng M [9]	+	-	-	+	+N	-	+	+
Hasegawa K [10]	NP	+	NP	-	+C	NP	+	NP
Petersson F [11]	+	NP	NP	NP	+N	NP	+	-
Kang W D [12]	+	-	NP	NP	NP	NP	+	NP
Guntupalli S [13]	NP	NP	NP	NP	NP	NP	+	NP
Hu, X [14]	+	NP	NP	NP	NP	NP	+	+
Hu, X [14]	+	-	-	+	NP	NP	NP	+
This case	+	-	-	+	-	+	-	+

NP: not performed; +C: Cytoplasmic positivity; +N: Nuclear positivity

### Pathological features

Morphologically, the tumor cells of ASPS are arranged in nested clusters or organ-like structures, and are spaced with slender fibrous blood vessels. The central cells of some tumor cell clusters are degenerated and poorly adherent, forming a characteristic pseudoadenoid structure. Characteristic immunohistochemical markers, such as *TFE3* positivity, are crucial for the diagnosis and differentiation of ASPS. In the current case, the immunophenotype showed strong nuclear staining for *TFE3*, which is consistent with previously reported cases, further supporting the diagnosis of ASPS.

*TFE3* (Transcription Factor E3) is a transcriptional enhancer located on the short arm of chromosome Xp11.23, encoding a protein that belongs to the microphthalmia-associated transcription factor (MiTF) family. In recent years, *TFE3* has gained prominence as an immunohistochemical marker for diagnosing ASPS. Notably, *TFE3* shares significant homology with MiTF, another member of this transcription factor family, which plays a crucial role in melanocyte development. The *TFE3* protein translocates to the nucleus when cells are under stress and/or starvation. *TFE3* gene fusions with various partner genes occur across a range of tumors, leading to nuclear expression of the *TFE3* protein. *TFE3* staining is not fully specific, as low nuclear expression of *TFE3* can be found in normal tissues. Nuclear *TFE3* positivity has also been observed in various tumors, including Xp11.2 translocation renal cell carcinoma, perivascular epithelioid cell tumors, and granular cell tumors. Therefore, additional immunohistochemical or molecular tests are required to accurately differentiate these diagnoses. *TFE3* was positive in 13 cases of cervical ASPS, including the present case, which confirms the high sensitivity of *TFE3* for the diagnosis of cervical ASPS.

A special stain for ASPS, PAS, is used to detect polysaccharides, especially glycogen, mucins, and certain glycoproteins, in tissue sections.ASPS is usually able to observe red rod or needle-like crystals in the cytoplasm. Among the 12 previously reported cases of cervical ASPS, 7 exhibited PAS-positive particles, while 5 cases did not undergo PAS staining. In the current case, PAS staining was negative, likely because the crystals had not yet fully formed and were only present as precursor granules. Tsuji et al. showed that that the differences in PAS staining results may be related to the way the samples were fixed or handled and the translocation of the *TFE3* gene, which may have led to abnormalities in the formation of PAS-positive crystals, and this explains the negative or weakly positive PAS in some cases. In Table 1, we summarize the PAS staining results from various studies, including the findings from Lee H J [8], who highlighted the variability in PAS positivity, emphasizing the influence of sample handling and genetic factors. While PAS-positive, diastase-resistant crystals were identified in 16 out of 21 cases (76%), these crystals were not prominently observed in the present case, where only poorly defined granules were found in the cytoplasm of the tumor cells. The table provides an overview of how PAS staining results differ across various ASPS cases, offering further insights into the complexity of PAS positivity in different contexts.

ASPS has long lacked specific and sensitive immunohistochemical markers. Early on, it was suggested that ASPS may originate from rhabdomyosarcoma due to the cytoplasmic expression of MyoD1 found in tumor cells of ASPS. Literature reports that approximately 50% of ASPS cases show MyoD1 positivity, with 40% positive for desmin and 20-30% focally positive for SMA [9]. However, MyoD1 is a specific marker for rhabdomyosarcoma, and its cytoplasmic expression in ASPS is often regarded as a false-positive due to variability in antibody clones. In our case, MyoD1 expression was assessed, and no significant cytoplasmic positivity was observed. The expression of desmin and SMA is also seen in other tumors, such as myofibroblastic tumors, osteoblastic fibromucinous tumors, and giant cell tumors of the tendon sheath, which further complicates the interpretation of these markers in ASPS. In this case, MyoD1 was negative and desmin and SMA were focally positive, which may represent a heterogeneous or incompletely differentiated state of the tumor cells. Regarding MyoD1 expression in our study, the results of the immunohistochemical testing, as summarized in Table 1, revealed that MyoD1 was negative for both cytoplasmic and nuclear staining in our case, as well as in several other reported cases of ASPS in the uterine cervix. This suggests that MyoD1’s cytoplasmic positivity, which has been observed in other studies, does not universally apply to all ASPS cases, highlighting the variability in its expression. In particular, the lack of MyoD1 expression in our case may suggest that MyoD1 positivity is not always associated with ASPS, and other markers should be considered in the differential diagnosis. This also emphasizes the importance of considering the heterogeneity of ASPS when interpreting MyoD1 expression patterns across different cases, as documented in the literature [15].

In most cases, epithelial markers (CK, EMA, etc.), neuroendocrine markers (CgA, Syn, S-100, etc.), and specific melanocyte markers like HMB45 and melan-A are typically negative, whereas nonspecific markers such as NSE and vimentin are often positive in a significant proportion of ASPS cases. The earliest hypothesis of neurogenicity in ASPS dates back to the 1950s, due to the fact that tumor cells show ultrastructurally similar features to the neuromuscular spindle. Although ASPS sometimes shows expression of some neurogenic markers (e.g. S-100 and NSE), which are occasionally positive in tumors, they are not prevalent or specific, which makes the neurogenic hypothesis lack strong support. This suggests that the origin of ASPS still needs to be further clarified.

ASPL refers to the *ASPSCR1-TFE3* fusion gene, triggered by the chromosomal translocation t(X;17)(p11;q25), which results in the rearrangement of the ASPSCR1 gene on chromosome 17 and the *TFE3* gene on the X chromosome. This fusion gene plays a key role in the pathogenesis of ASPS, but it is not an immunohistochemical stain. This translocation results in a fusion of the two genes, forming an important gene product that is tumorigenic in ASPS. The *ASPSCR1-TFE3* gene was first identified and reported by Ladanyi et al. [16] Schoolmeester et al. [6] identified the *ASPSCR1-TFE3* gene fusion in ASPS using FISH or reverse transcription polymerase chain reaction (RT-PCR). *ASPSCR1-TFE3* remains the most sensitive marker for diagnosing ASPS when detected by molecular testing, specifically through techniques such as PCR or FISH that identify the fusion gene. In our study, the positive expression rate of *ASPSCR1-TFE3* was 75% (6/8). It has been shown that *ASPSCR1-TFE3* is not only present in ASPS, but also in Xp11.2 translocation-associated renal cell carcinoma [17]. This molecular and histologic similarity has led some investigators to propose that ASPS may be more closely related to renal cell carcinoma, and some have suggested that ASPS is a renal cell carcinoma subtype. However, this hypothesis remains debated, as renal cell carcinomas with the Xp11.2 translocation can also harbor other *TFE3* fusion genes, like *PRCC-TFE3*. Currently, there is no definitive evidence to confirm that ASPS originates from nephrogenic tissue. The detection of the *ASPSCR1-TFE3* fusion gene is considered the gold standard for diagnosing ASPS, as it has extremely high sensitivity and specificity. This molecular test greatly reduces the likelihood of misdiagnosis and provides a reliable diagnostic criterion.

### Differential diagnosis

#### PEComa

Microscopically, the tumor cells are epithelioid and arranged around blood vessels in radial, petal-like, or sheet-like patterns. The surrounding stroma contains a rich network of thin-walled vessels. ASPS typically forms vesicles and nests, with an interconnected capillary network between the nests. There is histomorphologic overlap between ASPS and *TFE3*-rearranged PEComa, making it challenging to distinguish these entities based on morphology alone. Immunohistochemically, PEComa typically shows positivity for melanocytic markers such as HMB45 and Melan-A, in contrast to ASPS, which remains negative for these markers [18].

Both PEComa and ASPS involve rearrangements of the *TFE3* gene. ASPS is associated with an *ASPSCR1-TFE3* gene fusion, while PEComa typically exhibits an *SFPQ-TFE3* fusion. However, there are a few cases of PEComa that show the *ASPSCR1-TFE3* fusion. Interestingly, some PEComa cases are morphologically closer to typical PEComa than to ASPS [19]. It has been suggested that ASPS and *TFE3*-rearranged PEComa may represent different phenotypes of the same tumor spectrum [20], but its accuracy still needs to be confirmed by more subsequent studies. In this case, the initial pathological diagnosis was considered as PEComa, primarily due to the tightly arranged focal tumor nests with subtle loss of adhesion, small foci with few spindle cells, and a morphology resembling PEComa. However, negative staining for melanocytic markers, which are typically expressed in PEComa, led to further investigation. The diagnosis was ultimately confirmed by *ASPSCR1-TFE3* fusion gene testing.

#### Clear cell carcinoma

The tumor cells grow in a nested, tubular, or vesicular pattern; the cytoplasm is clear; the interstitium has a translucent, gel-like appearance; and the cells contain distinct nucleoli. The morphology is diverse and includes cystic, hemorrhagic, or degenerative scar-like components. *TFE3*-associated Xp11 translocations have also been observed in clear cell carcinoma and renal cell carcinoma. Besides the *ASPSCR1-TFE3* fusion gene, other detectable fusion genes include *PRCC-TFE3*, along with alterations in the VHL gene [21]. Identification was possible through the observation of typical morphological structures, and the immunohistochemical findings included PAX8 and CK positivity [22], while *ASPSCR1-TFE3* fusion gene testing showed mostly negative results. It is important to note that ASPS refers to the genetic fusion and is not an immunohistochemical stain.

#### Alveolar rhabdomyosarcoma

This is a malignant tumor characterized by the diffuse proliferation of small round cells. These cells morphologically overlap with various small round cell malignancies and are often associated with cytoplasmic vacuoles. The cells are predominantly arranged in nests and vesicles, with fibrovascular intervals between the vesicles. Common gene fusions, such as PAX3-FOXO1 (t(2;13)(q35;q14)) or PAX7-FOXO1, are frequently observed. Tumor cells are typically positive for Desmin, SMA, Myogenin, and MyoD1 [23], but negative for *TFE3*. While these features overlap with those of ASPS, the key distinction lies in the absence of *TFE3* positivity in this tumor, which is a hallmark of ASPS, as well as the gene fusion profiles that differ between the two entities.

### Treatment and prognosis

In cases where the tumor can be completely resected, effective resection of the lesion is the primary therapeutic modality for the treatment of ASPS. Relevant literature reports that radiation therapy as an adjuvant treatment failed to significantly improve the survival rate of patients, and the efficacy of chemotherapy and radiotherapy needs to be further confirmed [24, 25]. Targeted agents are increasingly used in tumor therapy, such as MET inhibitors and anti-angiogenic drugs. The generation of the *ASPSCR1-TFE3* fusion protein results in the expression of an activatable tyrosine kinase (MET) [26]. Tyrosine kinase inhibitors have shown efficacy in treating ASPS, particularly in cases with metastatic or inoperable tumors. Drugs like sunitinib, cediranib, and pazopanib have demonstrated response rates exceeding 50% [27]. These inhibitors target angiogenesis pathways, which are crucial in ASPS due to the tumor’s high vascularity. Large multicenter studies are still needed to explore the prognosis of targeted therapy versus immunotherapy. Younger patients with ASPS and those with smaller tumor diameters tend to have a more favorable prognosis. Among ASPS cases of the female genital tract, tumors located in the uterine corpus generally have the best prognosis, followed by those in the cervix, whereas perineal ASPS is associated with the poorest prognosis [7].

## Conclusion

In summary, ASPS is a tumor characterized by specific genetic fusions, with the most common primary sites being soft tissue locations, particularly the extremities. Although ASPS can occur in the female genital tract, such occurrences are relatively rare compared to its more frequent presentation in soft tissue sites. The disease most commonly affects young individuals, with female patients representing a significant proportion of cases. The cervix, vagina, and vulva are less commonly involved in comparison to the more frequent soft tissue sites such as the lower extremities, as supported by previous studies, including the SEER analysis by Wang et al. [4]. ASPS has a characteristic molecular phenotype, which helps to distinguish it from other diseases such as *TFE3* translocation-associated PEComa and clear cell carcinoma. More and more targeted drugs for the treatment of ASPS have been discovered in recent years, and their efficacy needs to be further evaluated.

## Data Availability

No datasets were generated or analysed during the current study.

## Abbreviations

ASPS: Alveolar Soft-Part Sarcoma
CgA: Chromogranin A
PEComa: Perivascular Epithelioid Cell Tumor
TFE3: Transcription Factor E3
PAS: Periodic Acid-Schiff Staining
MRI: Magnetic Resonance Imaging
LEEP: Loop Electrosurgical Excision Procedure
HPF: High-Power Field
EMA: Epithelial Membrane Antigen
SMA: Smooth Muscle Actin
RT-PCR: Reverse Transcription Polymerase Chain Reaction
FISH: Fluorescence In Situ Hybridization
ASPSCR1-TFE3: Alveolar Soft-Part Sarcoma Chromosomal Translocation Involving TFE3
